# Long-term outcomes of mechanochemical ablation using the Clarivein device for the treatment of great saphenous vein incompetence

**DOI:** 10.1016/j.jvsv.2024.101967

**Published:** 2024-09-11

**Authors:** Sharon Oud, Tamana Alozai, Yee Lai Lam, Çağdaş Ünlü, Michael Mooij, Michiel A. Schreve

**Affiliations:** a Department of Surgery, Amsterdam University Medical Centre, Amsterdam, The Netherlands; b Department of Surgery, Northwest Clinics, Alkmaar, The Netherlands; c Department of Surgery, Onze Lieve Vrouwe Gasthuis, Amsterdam, The Netherlands; d Department of Dermatology, Zaans Medical Centre, Zaandam, The Netherlands; e Department of Surgery, Red Cross Hospital, Beverwijk, The Netherlands; f Department of Phlebology, Skin and Vein Clinic Oosterwal, Alkmaar, The Netherlands

**Keywords:** Clarivein, Great saphenous vein, Mechanochemical ablation, Varicose veins, Venous insufficiency

## Abstract

**Objective:**

The short-term anatomical success rates of mechanochemical ablation using the Clarivein device (Merit Medical) in the treatment of great saphenous vein (GSV) incompetence are high. However, the anatomical success rates seem to drop over time. The aim of this study was to determine the long-term outcomes of GSV treatment using the Clarivein and to assess whether specific anatomical features better correlate with clinical or quality of life (QoL)-related outcomes.

**Methods:**

This is a single-center, prospective cohort study in follow-up of a multicenter, randomized controlled trial using Clarivein with liquid polidocanol for the treatment of GSV incompetence. The primary outcome was anatomical success (AS), defined as complete occlusion or a recanalized segment, irrespective of reflux, of <10 cm in length. In addition, reflux-free anatomical success (RF-AS) was determined, and defined as complete occlusion or a recanalized segment with <10 cm of reflux. Clinical success was assessed using the Venous Clinical Severity Score (VCSS), and QoL was assessed using the Dutch version of the Aberdeen Varicose Vein Questionnaire (DAVVQ) and the 36-Item Short Form Health Survey (SF-36). Subgroup analyses were performed based on whether AS or RF-AS was achieved or not.

**Results:**

A total of 109 patients (115 limbs) were included. The mean follow-up time was 8.4 ± 0.9 years (range, 5.5-10.3 years). AS was seen in 60.5% of limbs, and RF-AS was seen in 72.8% of limbs. Compared with baseline, the overall mean VCSS improved from 5.3 ± 2.4 to 4.1 ± 2.4, and the overall median DAVVQ score from 13.5 (interquartile range [IQR], 8.7-20.0) to 10.5 (IQR, 5.3-16.2) (*P* < .001). Improvement in VCSS was only significant in patients with successful treatment: from 5.5 ± 2.7 to 3.7 ± 2.5 (*P* < .001) if AS was achieved and from 5.0 ± 1.7 to 4.5 ± 1.9 (*P* = .20) if AS was not achieved. The same results were found for DAVVQ scores: improvement from13.5 (IQR, 8.7-20.6) to 10.3 (IQR, 3.0-14.5) (*P* < .01) if AS was achieved and from 12.9 (IQR, 8.3-19.3) to 10.8 (IQR, 6.7-18.2) (*P* = .35) if AS was not achieved. Regarding the overall SF-36 scores, the domains of vitality, mental health, and general health worsened significantly.

**Conclusions:**

In over 8 years of follow-up, the anatomical success rate after the treatment of GSV incompetence using the Clarivein device decreased to 60.5%. However, clinical scores and disease-specific QoL still improved significantly compared with baseline. We found no convincing evidence that the absence of reflux correlates better with clinical and QoL-related outcomes compared with recanalization irrespective of reflux.

**Article Highlights**

• **Type of Research:** Single-center prospective cohort study
• **Key Findings:** During an 8.4-year follow-up in 109 patients (115 limbs) treated for great saphenous vein incompetence using Clarivein, anatomical success was seen in 60.5% of limbs and reflux-free anatomical success in 72.8% of limbs. The overall Venous Clinical Severity Score and Dutch Aberdeen Varicose Vein Questionnaire improved significantly compared with baseline.
• **Take Home Message:** Anatomical success after treatment with Clarivein seems to steadily decrease over time, although clinical scores and disease-specific quality of life scores still improved significantly compared with baseline.

Results regarding anatomical success, clinical success and changes in quality of life (QoL) after mechanochemical ablation (MOCA) using the Clarivein device (Merit Medical) are limited to a follow-up period of 5 years.^1^, ^2^, ^3^

Although endovenous thermal ablation is the recommended first choice of treatment for (great) saphenous vein (GSV) incompetence, non-thermal, non-tumescent ablation is better tolerated by patients and has a lower risk of nerve injury.^4^^,^^5^ Previous studies have shown that treatment with Clarivein results in less pain both during and after treatment when compared with endovenous laser ablation (EVLA) or radiofrequency ablation (RFA).^5^, ^6^, ^7^, ^8^ Treatment with Clarivein is also associated with improved QoL and clinical success at 1 year of follow-up.^9^^,^^10^ The short-term (6-12 weeks) anatomical success rates are high, with vein occlusion rates of 90% to 97%.^11^, ^12^, ^13^ However, these success rates seem to drop over time. Witte et al conducted a systematic review analyzing outcomes after treatment of saphenous vein incompetence using Clarivein. The pooled anatomical success rate decreased from 92% after 6 months, to 91% after 1 year, and to 87% after 3 years.^13^ Only three studies reported outcomes after 5 years of follow-up and mentioned freedom of anatomical failure in 78.7% to 84.0%.^1^, ^2^, ^3^

Although these previous studies have shown a steady decrease in anatomical success rates but a persistent improvement in clinical success and QoL up to 5 years, it is unknown whether these results are maintained over a longer period of time. Also, the American Venous Forum and the Society of Interventional Radiology state that the exact anatomic results that correlate with improved long-term clinical outcomes have not been scientifically demonstrated yet.^14^

Therefore, the aim of this study was to determine the long-term outcomes of treatment of GSV incompetence with the Clarivein device and to assess whether specific anatomical features correlate better with clinical and QoL-related outcomes.

## Methods

### Study design

We conducted a single-center, prospective cohort study in follow-up of a multicenter, randomized controlled trial that aimed to identify the optimal dosage of polidocanol (POL) used for MOCA treatment with the Clarivein device (further referred to as the original trial).^15^ For logistic feasibility reasons, only patients treated at the Red Cross Hospital in the Netherlands were included in the current study. Patients were included between February 2022 and April 2023.

### Patients

The patient selection process has been previously published.^15^ In short, from 2012 to 2018, patients with symptomatic primary GSV incompetence, defined as retrograde flow lasting >0.5 seconds and measured in an upright position using duplex ultrasound (DUS), were included. Exclusion criteria were: age <18 years, previous surgery of the ipsilateral GSV, a GSV diameter >12.0 mm, obstruction of the deep venous system, a body mass index >40 kg/m^2^, and C5 and C6 Clinical, Etiologic, Anatomic, Pathophysiologic (CEAP) classification.^16^ Patients were randomized utilizing computer block randomization to be treated with Clarivein using either 2% or 3% POL. For patients with two affected limbs, each limb was randomized individually. The second limb was treated 1 to 11 months after the first treatment.

### Treatment

The patients included in this study were treated by one of two dedicated vascular surgeons specialized in the treatment of superficial venous pathology. Both surgeons performed a minimum of 10 supervised Clarivein treatments before treating study participants.

The treatment procedure has been previously described.^15^ In short, the area to be treated was prepared in a sterile fashion. The GSV was punctured under ultrasound guidance, and the Clarivein catheter was placed through a 4 Fr introducer sheet. The tip of the wire was positioned 2 cm distally from the saphenofemoral junction (SFJ), and the device was activated at a setting of 3500 rotations/min and held stationary for 3 seconds. Thereafter, the catheter was pulled back at a steady rate of 1 cm/6 seconds, with simultaneous infusion of a maximum dosage of 5 mL of 2% POL or 3% POL for a maximum length of 30 cm. No concomitant ambulatory phlebectomies were performed, and no additional phlebectomies were allowed within the follow-up period of the original trial (6 months). After the treatment, patients were advised to wear a class 2 thigh stocking continuously for 48 hours, followed by 2 weeks during daytime.

### Data collection

All patients treated at the Red Cross Hospital were consulted by telephone and asked if they wanted to participate in the follow-up study. Oral information was given, and a patient information folder was sent. If patients decided to participate, a cost-free follow-up visit was scheduled.

During the follow-up visit, a DUS of the GSV tract was performed with patients standing in an upright position and over a total length of 30 cm, starting at the SFJ. For each centimeter, it was determined whether the treated vein segment was occluded, recanalized (compressible, patent) without reflux, or recanalized with reflux. Reflux was again defined as retrograde flow lasting >0.5 seconds. Clinical success was evaluated using the Venous Clinical Severity Score (VCSS).^17^ Furthermore, patients were asked to complete two QoL questionnaires: the Dutch version of the Aberdeen Varicose Vein Questionnaire (DAVVQ) and the 36-Item Short Form Health Survey (SF-36).^18^^,^^19^ The patients’ medical files were reviewed to check if any ipsilateral interventions or reinterventions were performed between the end of the original trial and this follow-up study. Data from the original trial database was used to determine baseline characteristics and anatomical success (AS) at short-term follow-up (6 months) in this subgroup of patients.

## Primary and secondary outcomes

### Primary outcomes

The primary outcome was AS, defined as complete occlusion or a recanalized segment, irrespective of reflux, of <10 cm in length. To assess whether the absence of reflux better correlates with clinical and QoL-related outcomes, reflux-free anatomical success (RF-AS) was determined. RF-AS was defined as complete occlusion or a recanalized segment with <10 cm of reflux, meaning that the total length of the recanalized segment might be longer than 10 cm. For both definitions, treatment was deemed successful if AS or RF-AS was achieved and not successful if AS or RF-AS was not achieved. In patients who underwent a reintervention on the GSV between the end of the original trial and this follow-up study, treatment was deemed not successful.

### Secondary outcomes

Secondary outcomes included: clinical success (VCSS), disease-specific QoL (DAVVQ), generic QoL (SF-36), and ipsilateral (re)intervention rates.

### Statistical analysis

Data analyses were performed using SPSS 17 software (SPSS, Inc). Subgroup analyses were performed based on successful or unsuccessful treatment according to the AS and RF-AS definition. Of patients treated bilaterally, both limbs were excluded from analyses of the secondary outcomes of DAVVQ and SF-36 scores due to the inability to distinguish these outcomes for each limb separately. The primary outcomes (AS and RF-AS) were mentioned as frequencies and proportions. For continuous variables, within-group differences were analyzed using the paired *t*-test in case of normal distribution, or the Wilcoxon signed rank test in case of non-normal distribution. Between-group differences were analyzed using the unpaired *t*-test in case of normal distribution, or the Mann-Whitney *U* test in case of non-normal distribution. For categorical data, between group differences were analyzed using the χ^2^ test. Normally distributed data were reported as mean ± standard deviation (SD), and non-normally distributed data were reported as median with an interquartile range (IQR). A *P*-value of *P* < .05 was considered statistically significant.

### Ethical statement

This study was conducted following the principles outlined in the Declaration of Helsinki, adopted by the 18th World Medical Association General Assembly, Helsinki, Finland, June 1964, and subsequently amended.

The Medical Research Ethics Committee (MREC) of the Maastricht University Medical Center, that also approved of the original trial, reviewed the protocol, and confirmed that an official approval of this study by its committee was not required. All participants provided written informed consent prior to their inclusion in the study.

## Results

### Patient characteristics

One hundred nine of 170 patients (64.1%) treated at the Red Cross Hospital were included. Six of the included patients underwent bilateral treatment, resulting in a total of 115 (of 180) included limbs (63.9%). Reasons for exclusion were: unable to contact, deceased, cognitive or physical impairment, and refusal to participate (Table I).

**Table I:** Reasons for exclusion

Reasons for exclusion	Number of patients (number of limbs)
Unable to contact	17 (19)
Deceased^a^	15 (15)
Cognitive or physical impairment	7 (8)
Refusal to participate since no complaints	5 (5)
Refusal to participate for other reasons	17 (18)
Total excluded	61 (65)

a Malignancy, n = 9; pulmonary disease, n = 1; unknown, n = 5 (all of advanced age or with a complex medical history).

The mean follow-up time was 8.4 ± 0.9 years (range, 5.5-10.3 years). The mean age of patients was 59.5 ± 14.9 years, and 73.4% of patients (n = 80) were female. The POL concentration used was evenly distributed among the included limbs; 59 limbs were treated with 2% POL and 56 with 3% POL. Four of six patients that underwent bilateral treatment received different percentages of POL for each limb. Baseline characteristics are shown in Table II.

**Table II:** Baseline characteristics

Characteristic	Total population (n = 115 limbs)
Age, years	
At the moment of treatment	51.1 ± 15.0
At long-term follow-up	59.5 ± 14.9
Weight, kg	78.8 ± 13.8
Treated GSV diameter, mm	5.5 ± 1.1
Treated GSV length, cm (range)	29.5 ± 3.3 (18-31)
POL dosage used, mL	4.8 ± 0.5
POL percentage, n (%)	
2%	59 (51.3)
3%	56 (48.7)
Side branches, n	2.2 ± 1.6
CEAP C-classification, n (%)^a^	
C1	0 (0.0)
C2	2 (2.0)
C3	83 (79.0)
C4	20 (19.0)
Use of compression stocking, n (%)	33 (28.7)
Non-medical stockings	12 (10.4)
Medical stockings, not daily	11 (9.6)
Medical stockings daily	10 (8.7)

*CEAP*, Clinical-Etiology-Anatomy-Pathophysiology; *GSV*, great saphenous vein; *POL*, polidocanol.

Data are presented as mean ± standard deviation or number (%).

a CEAP data missing in 10 patients/limbs (8.7%).

## Primary outcomes

### Anatomical success

AS decreased from 88.5% (100/113) at short-term follow-up to 60.5% (69/114) at long-term follow-up. RF-AS was seen in 72.8% (83/114) at long-term follow-up. No significant differences were found in AS and RF-AS based on the concentration of POL used (*P* = .62 and *P* = .99, respectively). At short-term follow-up DUS data was missing in two patients and at long-term follow-up in one patient.

In 39.5% of limbs (n = 45), treatment was not successful. In 39 of these limbs, the vein was recanalized for at least 10 cm, two despite a reintervention on the GSV. In six limbs, a reintervention was performed, whereafter the vein remained occluded. The treated GSV length did not significantly differ between patients with successful or unsuccessful treatment (*P* = .131).

## Secondary outcomes

### Venous Clinical Severity Score (clinical success)

At long-term follow-up, the overall mean VCSS improved from 5.3 ± 2.4 to 4.1 ± 2.4 (*P* < .001). Table III shows differences in mean VCSS within subgroups based on successful or unsuccessful treatment according to the AS and RF-AS definition. Improvement in mean VCSS was only significant in patients in whom AS or RF-AS was achieved (5.5 ± 2.7 to 3.7 ± 2.5 and 5.4 ± 2.6 to 3.8 ± 2.4 [*P* < .001], respectively).

**Table III:** Differences in Venous Clinical Severity Score (*VCSS*) and Dutch Aberdeen Varicose Vein Questionnaire (*DAVVQ*) scores based on successful and unsuccessful treatment: between baseline and long-term follow-up within subgroups (from left to right), and at baseline and long-term follow-up between subgroups (from top to bottom)

Treatment success	Baseline	VCSS	*P*-value	Baseline	DAVVQ	*P*-value
		Long-term follow-up			Long-term follow-up	
Total population	5.3 ± 2.4	4.1 ± 2.4	< .001	13.5 (8.7-20.0)	10.5 (5.3-16.2)	< .001
AS achieved	5.5 ± 2.7	3.7 ± 2.5	< .001	13.5 (8.7-20.6)	10.3 (3.0-14.5)	< .001
AS not achieved	5.0 ± 1.7	4.5 ± 1.9	.20	12.9 (8.3-19.3)	10.8 (6.7-18.2)	.35
*P*-value	.34	.083		.66	.13	
RF-AS achieved	5.4 ± 2.6	3.8 ± 2.4	< .001	13.5 (8.1-20.6)	10.2 (4.6-15.4)	< .001
RF-AS not achieved	5.1 ± 1.9	4.8 ± 1.8	.44	13.0 (10.0-18.7)	11.8 (7.2-19.0)	.34
*P*-value	.58	.025		.98	.11	

*AS*, anatomical success; *RF-AS*, reflux-free anatomical success.

Data are presented as mean ± standard deviation or median (interquartile range).

Limbs (n = 12) of patients treated bilaterally (n = 6) were excluded from analyses of DAVVQ scores.

Table III also shows differences in mean VCSS at baseline and differences in mean VCSS at long-term follow-up between subgroups based on successful or unsuccessful treatment. The only significant difference was seen in mean VCSS at long-term follow-up between patients in whom RF-AS was achieved vs those in whom RF-AS was not achieved (3.8 ± 2.4 vs 4.8 ± 1.8 [*P* = .025], respectively).

#### DAVVQ scores

At long-term follow-up, the overall median DAVVQ score improved from 13.5 (IQR, 8.7-20.0) to 10.5 (IQR, 5.3-16.2) (*P* < .001). Table III shows differences in median DAVVQ scores within subgroups based on successful or unsuccessful treatment according to the AS and RF-AS definition. Improvement in median DAVVQ scores was only significant in patients in whom AS or RF-AS was achieved (13.5 [IQR, 8.7-20.6] to 10.3 [IQR, 3.0-14.5] and 13.5 [IQR, 8.1-20.6] to 10.2 [IQR, 4.6-15.4] [*P* < .001], respectively).

Table III also shows differences in median DAVVQ scores at baseline and differences in median DAVVQ scores at long-term follow-up between subgroups based on successful or unsuccessful treatment. There were no significant differences found.

#### SF-36 scores

At long-term follow-up, the domains of role physical, vitality, mental health, and general health worsened; the domains of physical functioning, role emotional, and social functioning remained the same, and the domain of bodily pain improved (Fig). Changes in the domains of vitality, mental health, and general health were statistically significant (all *P* < .001).

**Fig:**
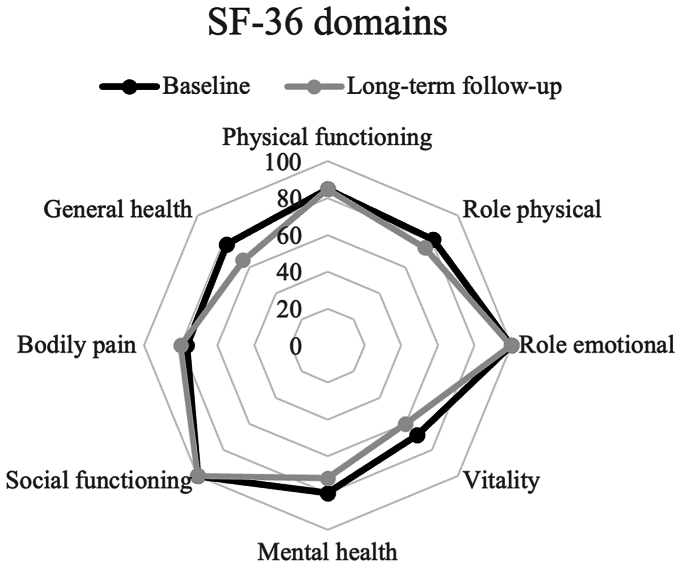
36-Item Short Form Health Survey (*SF-36*) domain scores at baseline (*black*) and long-term follow-up (*gray*). Limbs (n = 12) of patients treated bilaterally (n = 6) were excluded from analysis.

Differences in median SF-36 domain scores between subgroups based on successful and unsuccessful treatment according to the AS and RF-AS definition can be found in the Supplementary Table (online only). The only significant difference was seen in the domains of role physical and general health. These domain scores were significantly worse in patients in whom AS was achieved (*P* = .009 and *P* = .016, respectively).

### Ipsilateral (re)intervention rates

During the follow-up period, a total of 39 ipsilateral interventions were performed in 29 patients (Table IV), meaning some patients received multiple additional treatments. Of those 29 patients, eight patients, comprising eight limbs (7.0%), underwent a reintervention on the GSV after treatment failure. Five patients were treated with endovenous ablation, two with high ligation and stripping, and one patient was treated with ultrasound-guided foam sclerotherapy. The mean time to reintervention on the GSV was 40 ± 18.0 months.

**Table IV:** Overview of ipsilateral treatments performed during the follow-up period in n = 29 patients

Type of additional ipsilateral (re)interventions	Number of limbs
GSV (reinterventions)	
MOCA (Clarivein)	3
Endovenous laser ablation	1
Radiofrequency ablation	1
High ligation and stripping	2
Foam sclerotherapy	1
AASV	
MOCA (Clarivein)	3
Endovenous laser ablation	4
Phlebectomy	2
Foam sclerotherapy	1
Tributaries	
Phlebectomy	4
Foam sclerotherapy	8
SSV	
Radiofrequency ablation	1
Foam sclerotherapy	1
Giacomini vein: foam sclerotherapy	1
Perforating veins: foam sclerotherapy	6
Total	39

*AASV*, anterior accessory saphenous vein; *GSV*, great saphenous vein; *MOCA*, mechanochemical ablation; *SSV*, small saphenous vein.

## Discussion

This paper reports results of MOCA using the Clarivein device with a mean follow-up period of 8.4 years. AS was seen in 60.5% of limbs, but RF-AS was higher (72.8%). Despite this apparent drop in AS over time, clinical and QoL scores still improved significantly compared with baseline, especially in patients with successful treatment.

In this subgroup of patients treated at the Red Cross Hospital, the overall AS rate at 6 months was slightly lower compared with other short-term data on Clarivein (90%-94%).^13^ As discussed in the original trial paper, explanations for the relatively high rate of failure include a high contribution of limbs with a C3 and C4 CEAP classification, a maximum treated vein length of 30 cm, and a maximum volume of POL that could be used.^15^^,^^20^ Also, sotradecol is shown to be a more aggressive sclerosants compared with POL, which might yield better results.^21^ However, sotradecol is not registered for use in the Netherlands.

Nonetheless, this study indicates a further decrease in AS rates over time after treatment with Clarivein. Pooled anatomical success rates at 1, 2, and 3 years are reported to be 92%, 91%, and 87%, respectively.^13^ Three studies report outcomes after 5 years and mention freedom from anatomical failure in 78.7% to 84.0%.^1^, ^2^, ^3^ However, in this study, absolute numbers were used. When converting freedom from anatomical failure percentages from the previously mentioned studies to absolute numbers for comparison, anatomcial success rates would be 62.7% and 79.8%.^2^^,^^3^ Due to different methods of calculating anatomical success rates and also different definitions used to define anatomical success or anatomical failure, comparisons should be made with caution. This also applies when comparing these results with other frequently used endovenous treatment modalities. Hamman et al conducted a meta-analysis of randomized controlled trials with a follow-up of ≥5 years and showed a pooled anatomical success rate of 88% after EVLA, 83% after high ligation and stripping, and 34% after ultrasound-guided foam sclerotherapy.^22^ These data suggest that long-term anatomical success after Clarivein seems to be inferior to open surgical and endovenous thermal ablation techniques, the latter being currently recommended as first-line treatment for GSV incompetence,^4^ but still superior to foam sclerotherapy alone.

The natural progression of venous incompetence is likely to contribute to the steady decrease in anatomical success rates, as superficial venous incompetence is a chronic disease. Disease progression of venous incompetence is seen in over 50% of patients after more than 10 years of follow-up and the high incidence of ipsilateral interventions supports this statement.^23^^,^^24^ Previous literature also describes that most anatomical failures after Clarivein treatment occur in the first post-procedural year, which is suggested to be technique-related.^1^, ^2^, ^3^ This might explain why we found no significant difference in AS rates between the 2% POL and 3% POL group, whereas this difference was significant in the original trial.^15^ It might be that a higher concentration of POL leads to better outcomes in the short term and is thus technique-related, whereas long-term results primarily depend on other factors.

As for the secondary outcomes, the improvement in overall VCSS and DAVVQ scores, despite the deterioration in AS, is consistent with findings of other studies.^2^^,^^25^^,^^26^ However, a significant improvement in VCSS and DAVVQ scores was only found in patients with successful treatment. Our findings contradict earlier research on short- and long-term data, in which both patients with and without successful treatment showed significant improvement in VCSS and DAVVQ scores.^20^^,^^25^ Of note, the lack of significance in our study could be the result of a smaller sample size than both previously mentioned studies. Two Dutch papers report that the greatest improvements in VCSS and DAVVQ scores were seen after 1 to 3 years, whereafter these scores started to deteriorate again.^2^^,^^3^ Others report a steady improvement over time.^25^ Although we are not able to demonstrate the course of change for these scores, the median time to reintervention was approximately 40 months, consistent with previous literature.^27^ This probably resembles the moment of reocurrence of symptomatic varicose veins, which is likely to negatively influence clinical and disease-specific QoL scores.

We found no convincing evidence that the absence of reflux better correlates with clinical or QoL-related outcomes compared with recanalization irrespective of reflux. An explanation for the absence of reflux in a recanalized, compressible segment could be a reduction in vein diameter, restoring unidirectional flow as the result of increased vascular resistance and decreased flow velocity. Jin et al described that despite occlusion failure after RFA, significant reduction of saphenous vein diameter and loss of venous reflux were noted.^28^ A second explanation might be that near-normal physiological flow is restored by interrupting ‘escape points’ and eliminating venous-venous shunts, which is the same principle the ‘CHIVA method’ is based on.^29^ Improvements in VCSS and DAVVQ scores were significant in both patients in whom AS or RF-AS was achieved. The only difference that was found between patients with successful or unsuccessful treatment, was that in long-term VCSS scores according to the RF-AS definition. Hence, the lack of significance regarding the other outcomes could also be the result of a relatively small sample size. No other studies report clinical outcomes based on varying definitions of anatomical success, but there is conflicting evidence regarding long-term outcome differences between patients with successful and unsuccessful treatment. One study that defined anatomical success irrespective of reflux, reported a favorable difference in AVVQ scores, but not in VCSS, for non-recurrent patients compared with recurrent patients at 5-year follow-up.^25^ Another study did assess reflux and found significant worse VCSS and AVVQ scores in patients with remaining reflux after 1 year of follow-up.^30^ Still, there remains a gap in evidence regarding the link between anatomical and clinical outcomes. It might be worth assessing whether other quantifiable measurements, such as calf muscle pump function, provide a better correlation.

Apart from VCSS and DAVVQ scores, SF-36 scores were assessed as a measure of general health-related QoL. Because this is no disease-specific QoL tool, it is likely that changes in different domains are not solely related to superficial venous incompetence, but also to other health problems that occur with increasing age, especially in this predominantly female, more aged population (menopause, for example). This is reflected in the results, since most physical- and mental-related domains worsened at long-term follow-up. Our findings suggest that in the light of a worsened general health-related QoL, disease-specific QoL still improved.

### Study limitations and strengths

This study has several limitations. The main limitation is the relatively small sample size. We only included patients treated at the Red Cross Hospital in the original trial, and 64.1% of all eligible patients returned. This might have led to inclusion bias. Another limitation is that neovascularization and neo-reflux have not been assessed. These are well-described causes for recurrent varicose veins and might interfere with clinical outcomes.^31^ A third limitation is that deterioration in most SF-36 domains may have other causes in this aging population, unrelated to superficial venous incompetence, as previously mentioned. At last, changes in the clinical and QoL-related outcomes do not merely reflect the effect of the primary treatment, but also the effect of additional ipsilateral venous interventions performed between the end of the original trial and this follow-up study, and the use of compression stockings, as 28.7% of patients did in this cohort.

The major strength of our study is that it is the first one to report outcomes of Clarivein with a follow-up period of over 5 years. Another strength is that this is the first study to distinguish clinical and QoL-related outcomes based on different definitions of anatomical success.

## Conclusion

This study shows that in over 8 years of follow-up, AS after treatment of GSV incompetence using the Clarivein device seems to steadily decrease. However, clinical scores and disease-specific QoL scores still improved significantly compared with baseline. AS rates are higher when using the absence of reflux as a criteria of treatment success, but we found no convincing evidence that the absence of reflux better correlates with clinical and QoL-related outcomes compared with recanalization irrespective of reflux. Therefore, the gap in evidence regarding the correlation between anatomical and clinical outcomes remains. Because anatomical success seems to be inferior to endovenous thermal ablation techniques, Clarivein may be considered in a selected group of patients with a specific preference for this type of MOCA treatment.

## Author Contributions

Conception and design: TA, YL, MS

Analysis and interpretation: SO, CU, MM

Data collection: SO, TA

Writing the article: SO

Critical revision of the article: TA, YL, CU, MM, MS

Final approval of the article: SO, TA, YL, CU, MM, MS

Statistical analysis: SO

Obtained funding: Not applicable

Overall responsibility: SO

## Funding

None.

## Disclosures

None.
