# Supplementary material for: Long-term outcomes of mechanochemical ablation using the Clarivein device for the treatment of great saphenous vein incompetence
Source: J Vasc Surg Venous Lymphat Disord. 2024 Sep 11;13(1):101967. doi: 10.1016/j.jvsv.2024.101967 (PMC11764119; doi:10.1016/j.jvsv.2024.101967)
Supplement: Supplemental Table [file mmc1.docx]

# Supplemental Tables

Supplemental Table I: Differences in SF-36 domain scores at long-term follow-up between subgroups based on successful and unsuccessful treatment

| **SF-36 domains** | **AS achieved** | **AS not achieved** | **p-value** | **RF-AS achieved** | **RF-AS not achieved** | **p-value** |
| --- | --- | --- | --- | --- | --- | --- |
| Median PF score | 85.0 (65.0-95.0) | 90.0 (65.0-96.3) | .38 | 85.0 (65.0-100.0) | 90.0 (62.5-95.0) | .64 |
| Median RP score | 68.8 (43.8-100.0) | 90.3 (71.9-100.0) | .009 | 75.0 (50.0-100.0) | 87.5 (50.0-100.0) | .27 |
| Median RE score | 91.7 (58.3-100.0) | 100.0 (72.9-100.0) | .074 | 100.0 (66.7-100.0) | 100.0 (66.7-100.0) | .64 |
| Median VT score | 60.0 (50.0-70.0) | 65.0 (55.0-70.0) | .18 | 60.0 (50.0-70.0) | 60.0 (54.2-70.0) | .62 |
| Median MH score | 72.0 (56.0-76.0) | 72.0 (64.0-80.0) | .14 | 72.0 (60.0-76.0) | 68.0 (62.0-80.0) | .72 |
| Median SF score | 87.5 (62.5-100.0) | 100.0 (71.9-100.0) | .34 | 93.8.5 (65.6-100.0) | 100.0 (62.5-100.0) | .77 |
| Median BP score | 77.5 (57.5-90.0) | 80.0 (67.5-100.0) | .14 | 77.5 (67.5-100.0) | 80.0 (67.5-90.0) | .90 |
| Median GH score | 60.0 (45.0-75.0) | 77.5 (55.0-81.3) | .016 | 62.5 (45.0-80.0) | 70.0 (50.0-80.0) | .92 |

*BP,* bodily pain*; GH;* general health*; MH,* mental health*; PF,* physical functioning*; RE,* role emotional*; RP,* role physical*; SF,* social functioning*; VT,* vitality*.*

Data are presented as mean ± standard deviation or median (interquartile range).

Limbs (n=12) of patients treated bilaterally (n=6) were excluded from analyses.
